# Supplementary material for: Manipulating the EphB4-ephrinB2 axis to reduce metastasis in HNSCC
Source: bioRxiv. 2024 Jul 23:2024.07.21.604518. Preprint. [Version 1] doi: 10.1101/2024.07.21.604518 (PMC11291065; doi:10.1101/2024.07.21.604518)

Supplemental Figure 1: Loss of EphB4 in cancer cells significantly increases local tumor growth in the context of radiotherapy.

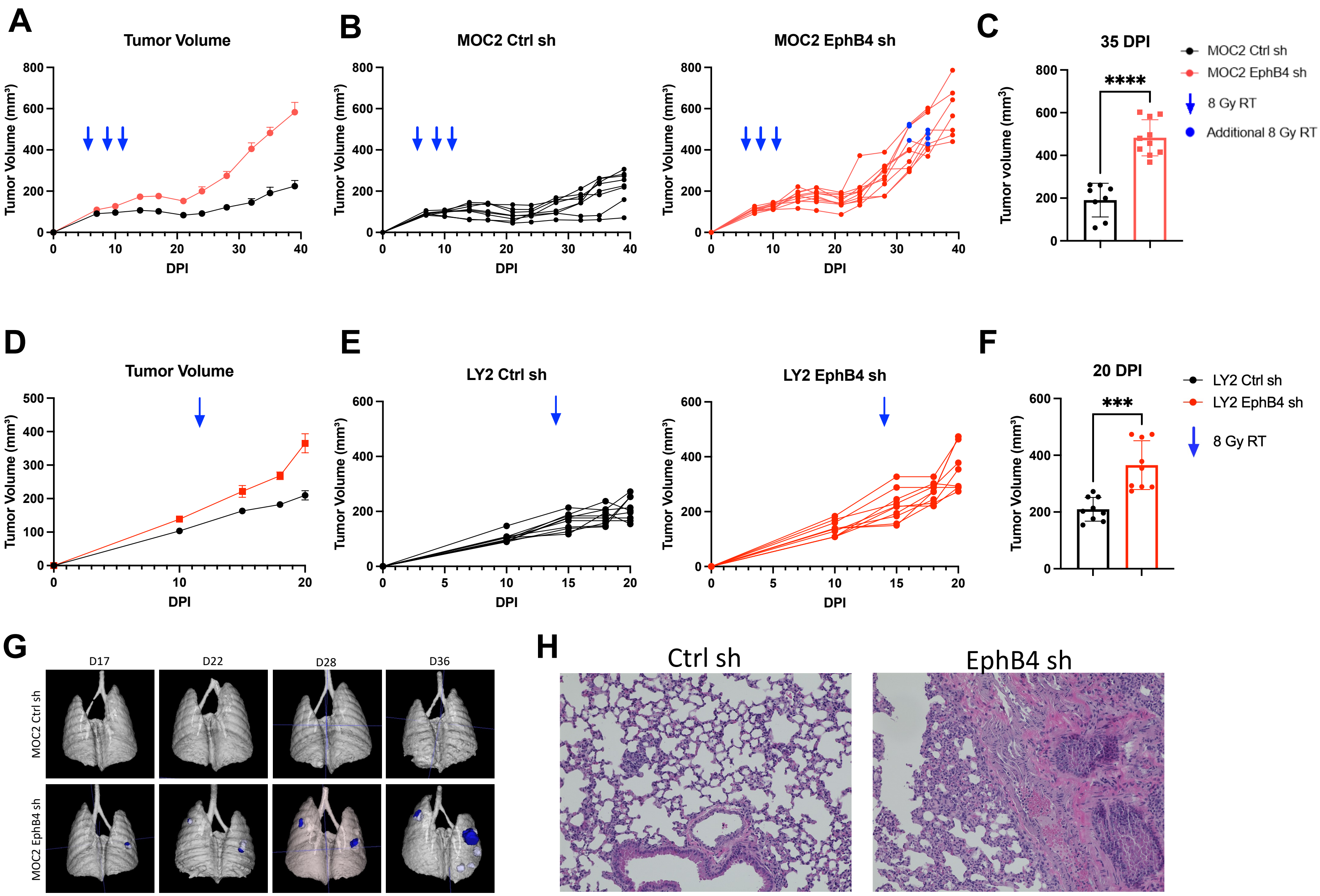

Supplemental Figure 2 : Gating strategy for cancer cell and CD4+ T cell coculture experiment

A **Gating strategy**

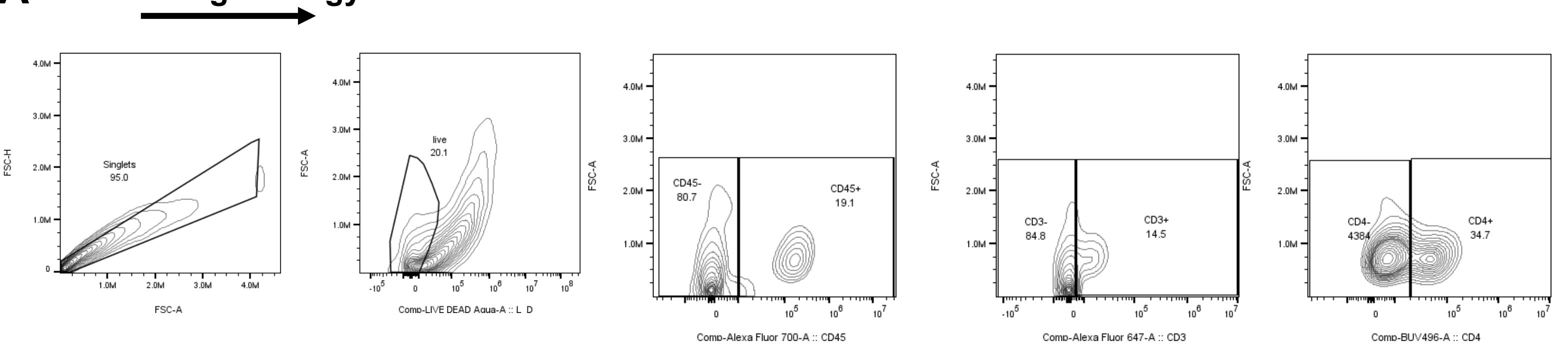

Supplemental Figure 3: Tregs from EphB4 KD tumors downregulate Th1 and Th2 differentiation and mTOR signaling *in vivo*.

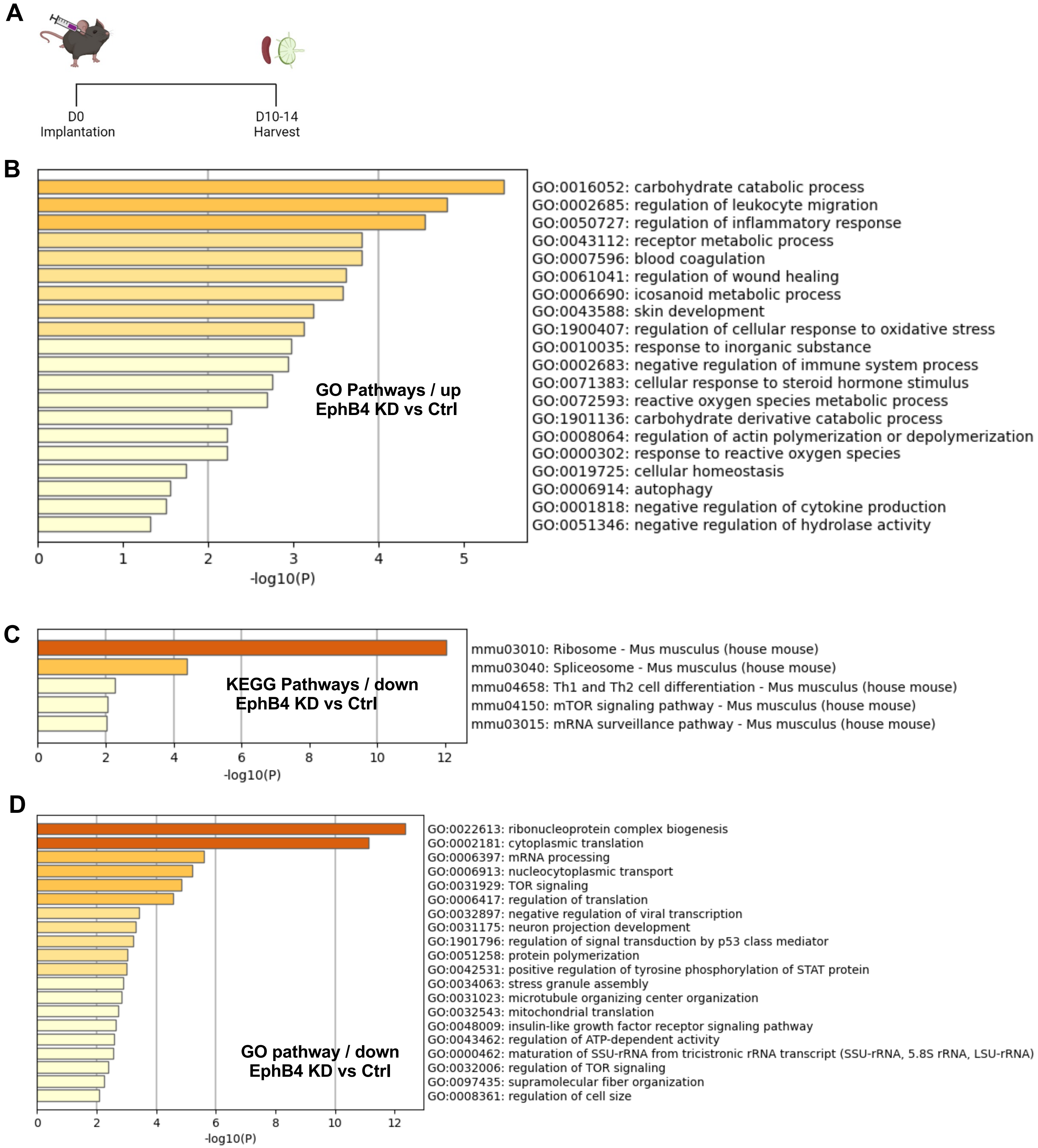

Supplemental Figure 4: EphrinB2 KO in vascular endothelial cells coupled with radiation therapy reduces local tumor growth.

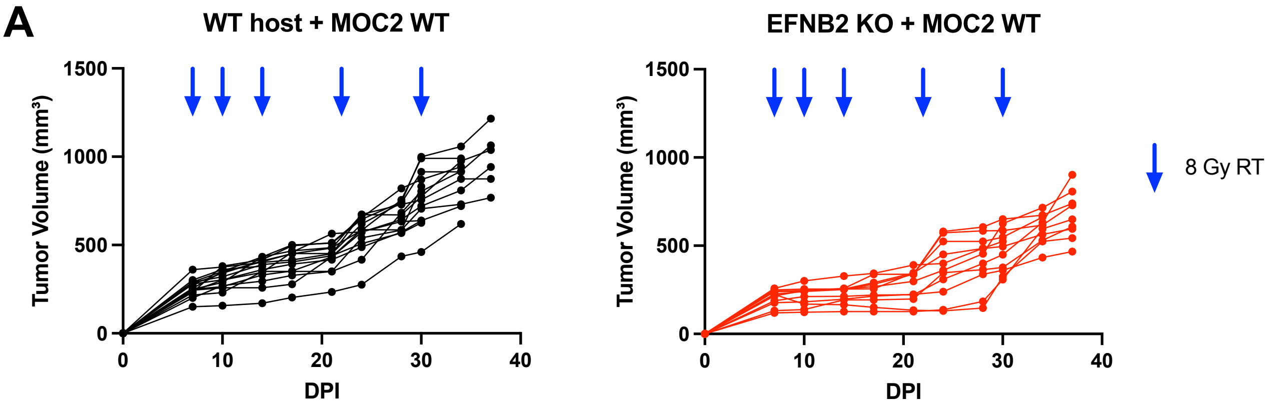

Supplemental Figure 5: EphrinB2 expression in vascular endothelial cells increases after RT.

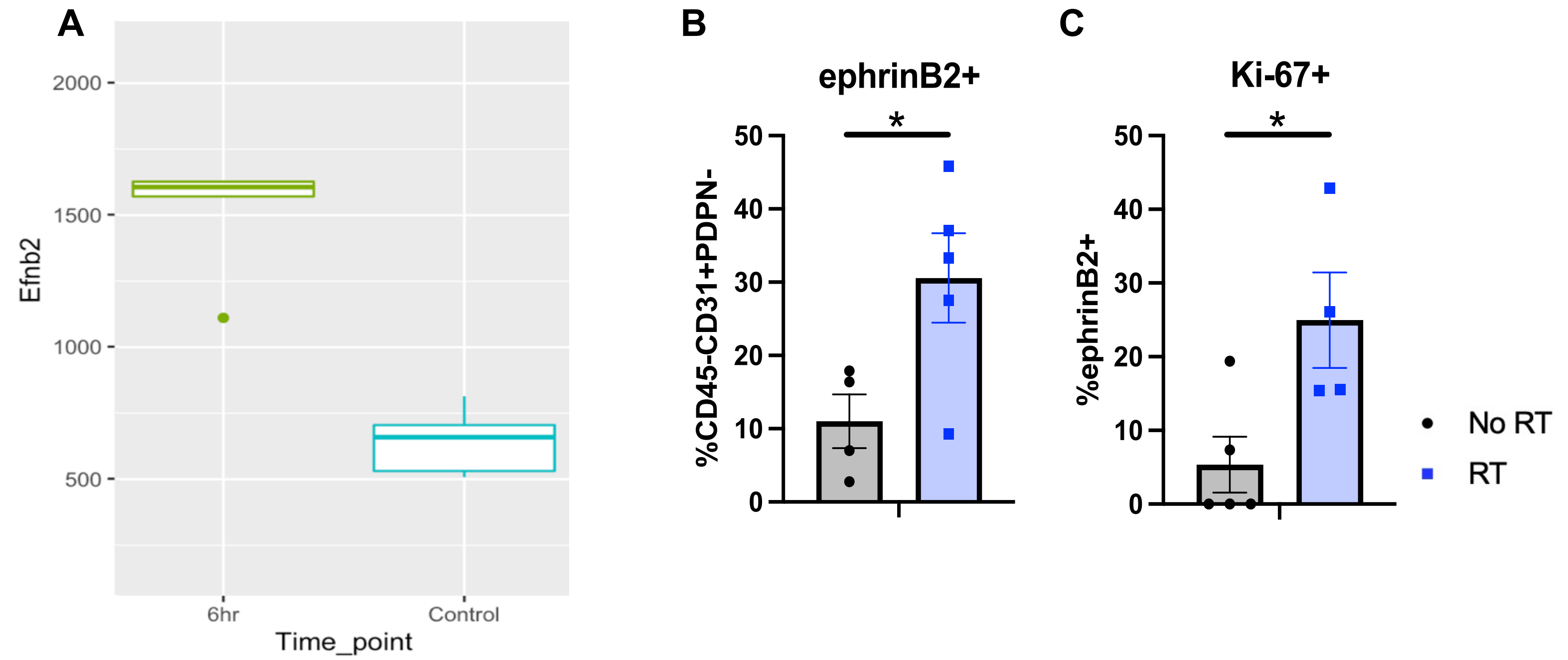

Supplemental Figure 6: Gating strategy for immune cell populations in the TME of ephrinB2 KO mice.

A

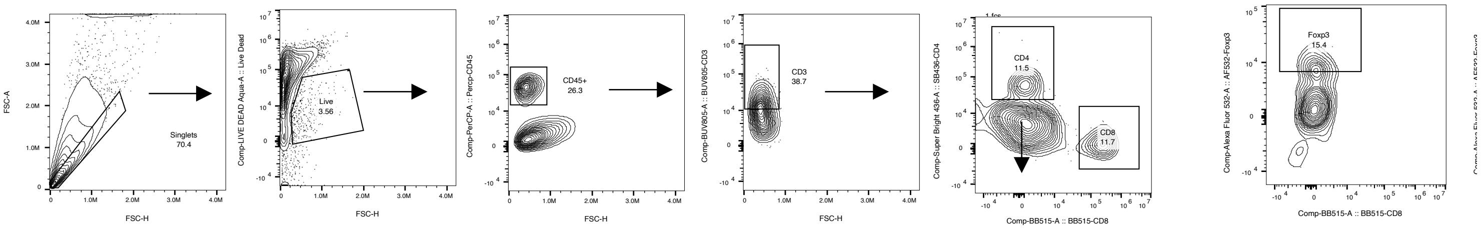

Supplemental Figure 7: EphrinB2 KO in vascular endothelial cells affects the systemic immune response.

A **Blood gating**

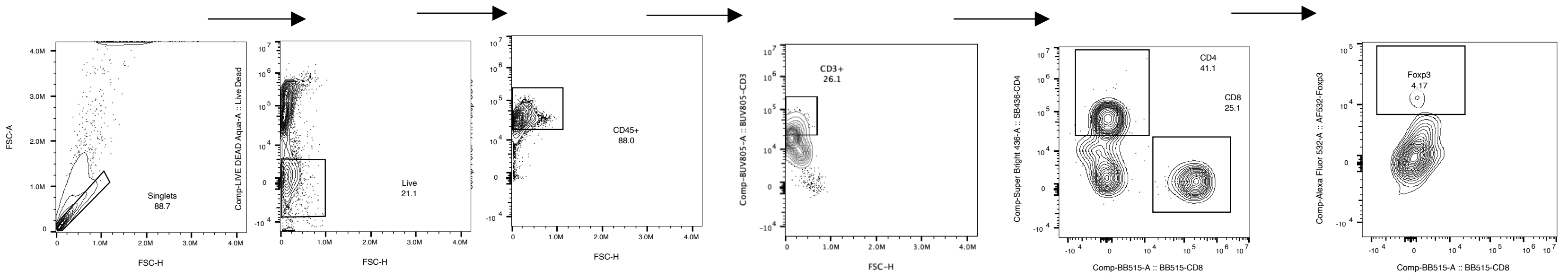

B

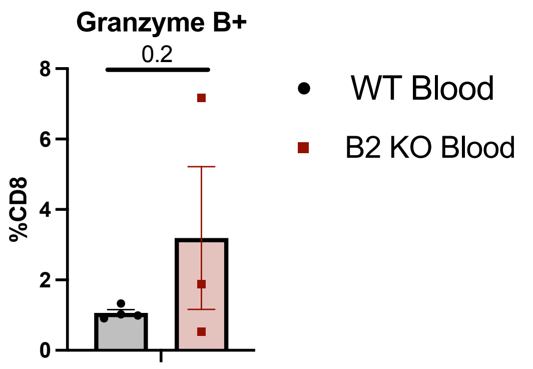

C **DLN gating**

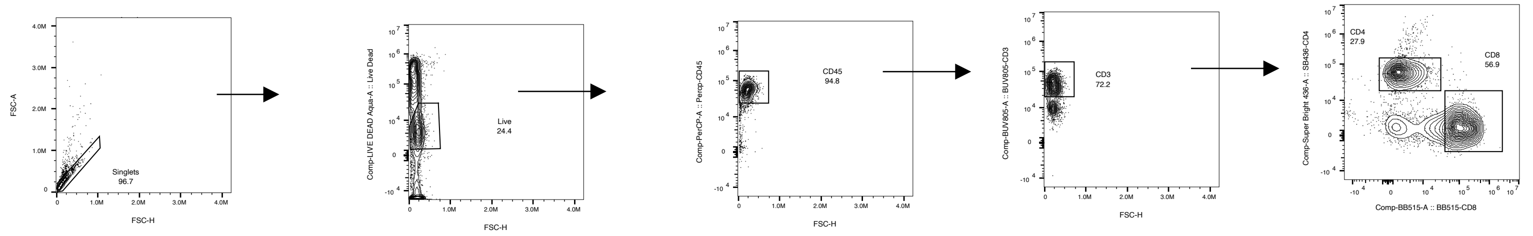

D **E** **F** **G**

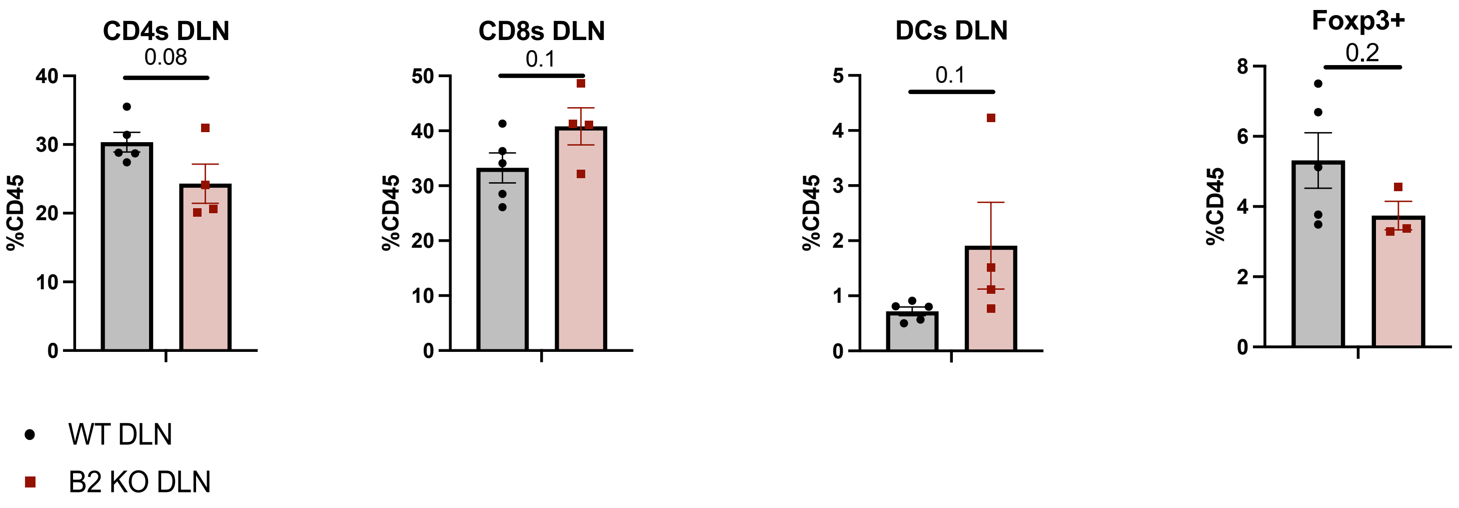

Supplemental Figure 8: Specialized flow cytometry demonstrates increased CD4 T cell accumulation in the TME of ephrinB2 KO mice.

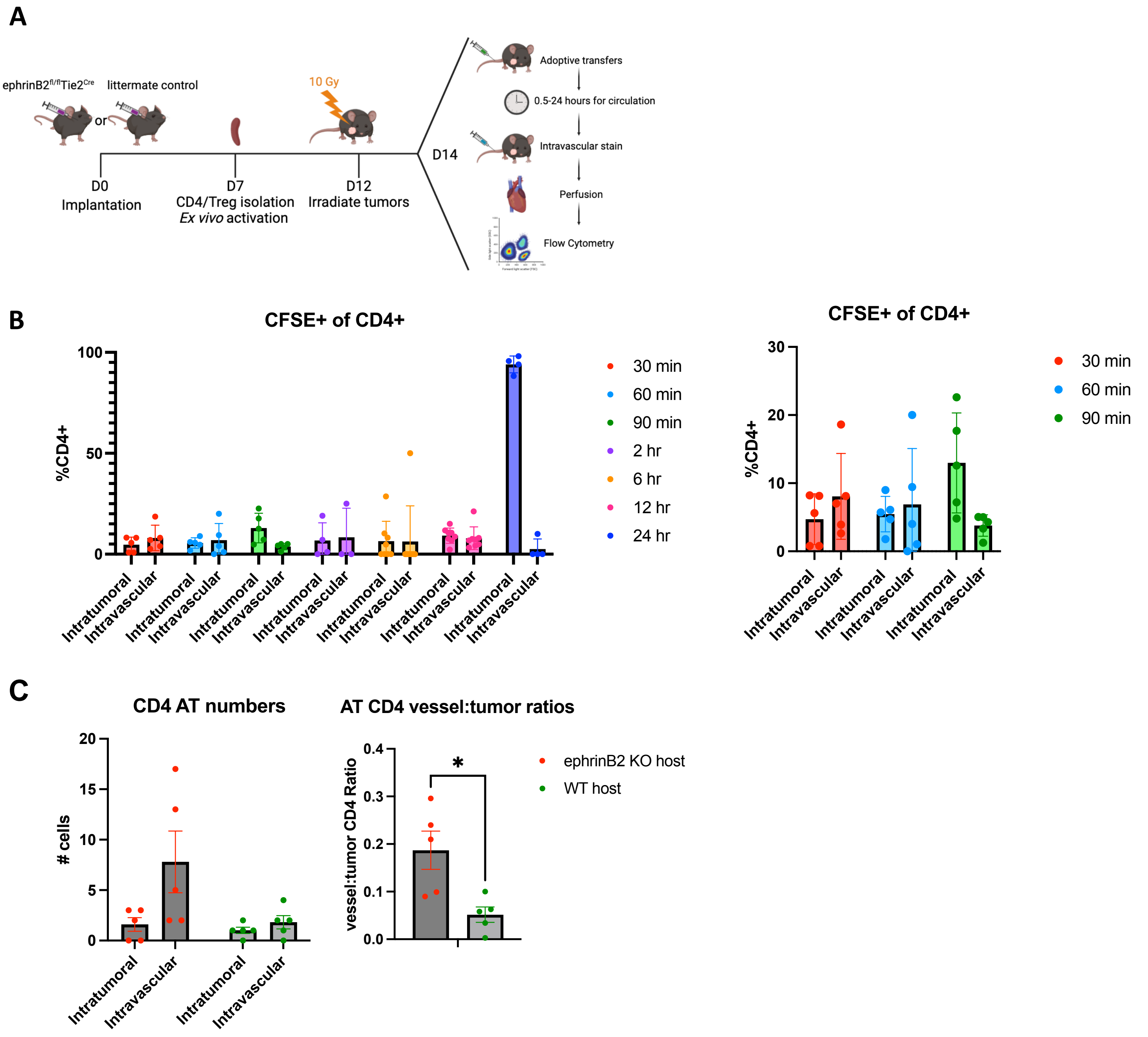

Supplemental Figure 9: EFNB2-Fc-His and Fc-TNYL-RAW-GS reduce local tumor growth.

A

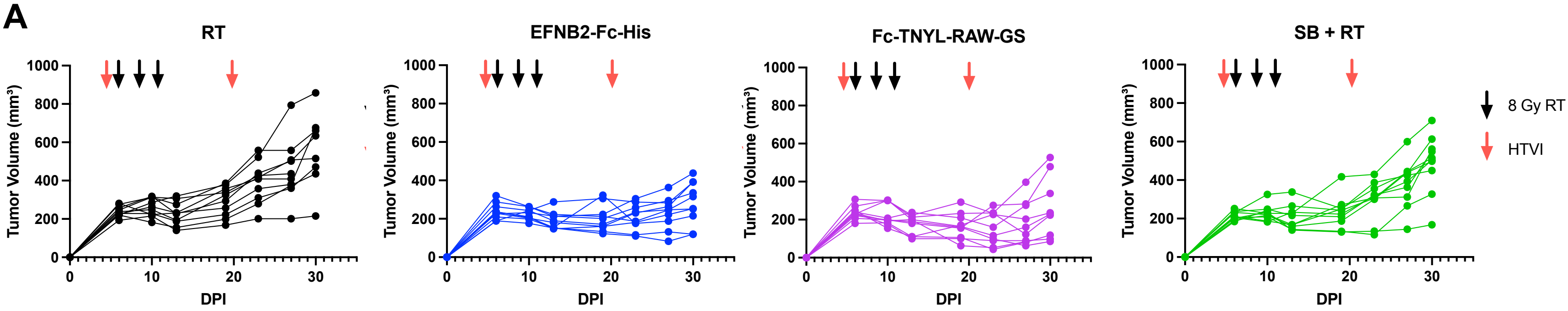

Supplemental Figure 10: Detection of Fc fusion proteins in mouse serum and EphB4 phosphorylation following treatment with EFNB2-Fc-His and Fc-TNYL-RAW-GS.

A

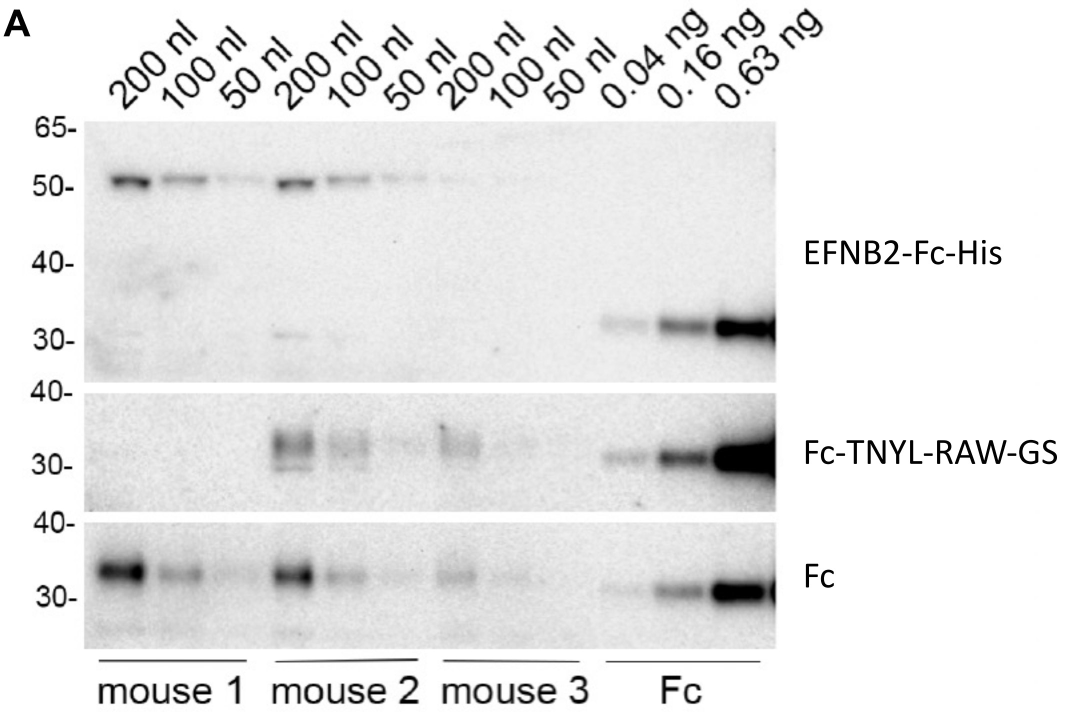

B

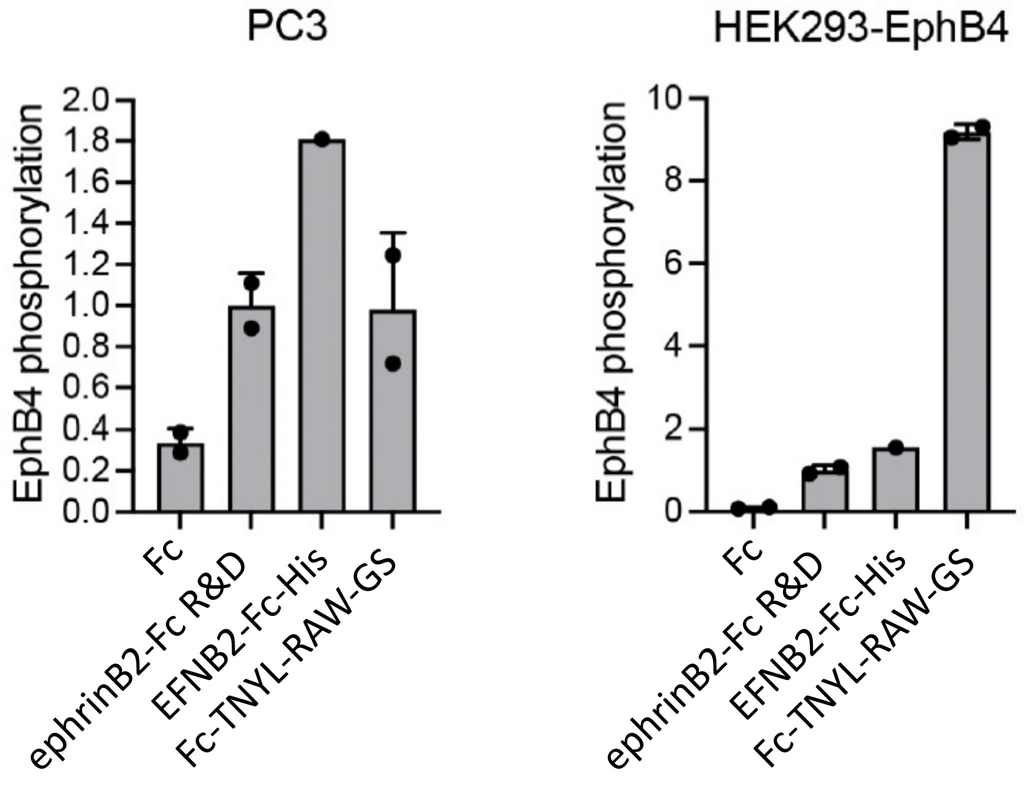

Supplement: Supplement 1 — Supplemental Figure 1: Loss of EphB4 in cancer cells significantly increases local tumor growth in the context of radiotherapy. (A) Average tumor volume curves comparing Ctrl shRNA versus EphB4 shRNA MOC2 tumors implanted in C57BL/6 mice. (B) Spaghetti plots of tumor volume for C57BL/6 mice implanted with MOC2 Ctrl (black) or EphB4 shRNA (red) tumors. (C) Dot plot showing significantly larger average tumor volume at 35 DPI in MOC2 implanted C57BL/6 mice. (D) Average tumor volume curves comparing Ctrl shRNA versus EphB4 shRNA LY2 cancer cells implanted in BALB/c mice. (E) Spaghetti plots of tumor volume for BALB/c mice implanted with LY2 Ctrl (black) or EphB4 shRNA (red) tumors. (F) Dot plot showing significantly larger average tumor volume at 20 DPI in LY2 EphB4 shRNA implanted BALB/c mice. (G) Representative 3D lung contouring generated using ITK-Snap from CT scans collected over time for MOC2 implanted C57BL/6 mice. Lung lesions are shown in blue. (H) Hematoxylin and eosin staining of lung tissue for Ctrl and EphB4 shRNA MOC2 implanted C57BL/6 mice. Comparison of tumor volume between the control and experimental group was done using a two-sided student’s t-test. Significance was determined if the p-value was <0.05*, <0.01**, <0.001***, and <0.0001****. p-values are indicated for the figures C ****p < 0.0001, F *p = 0.0002. Supplemental Figure 2: Gating strategy for cancer cell and CD4+ T cell coculture experiment. (A) Gating strategy for flow conducted after coculture of LY2 Ctrl or EphB4 shRNA cancer cells with CD4+ T cells. Supplemental Figure 3: Tregs from EphB4 KD tumors downregulate Th1 and Th2 differentiation and mTOR signaling in vivo. (A) Schematic experimental design. Tregs were isolated from spleens and lymph nodes using a MACS Miltenyi Biotec CD4+CD25+ Regulatory T Cell Isolation Kit, and cells were subsequently flow sorted by CD25+ expression. B) Bulk proteomic pathway analyses showing GO processes that were upregulated in Tregs of MOC2 EphB4 shRNA com [file media-1.pdf]
